# E-leadership in distributed new product development teams: unveiling the interplay between performance, team emotional capability, and engagement

**DOI:** 10.3389/fpsyg.2026.1802949

**Published:** 2026-06-16

**Authors:** Ayşe Günsel, Büşra Tekin, Hüseyin Rıdvan Yurtseven, Oktay Koç

**Affiliations:** 1 Business Administration, Faculty of Business Administration, Kocaeli University, Kocaeli, Türkiye; 2 Healthcare Management, Faculty of Health Sciences, University of Health Sciences, İstanbul, Türkiye; 3 Business Administration, Faculty of Economics, Administrative and Social Sciences, İstanbul Topkapı University, İstanbul, Türkiye

**Keywords:** distributed teams, e-leadership, team emotional capability, team engagement, team performance

## Abstract

**Introduction:**

Distributed work has made e-leadership central to how teams coordinate, connect, and perform through digital technologies. This is particularly important in distributed new product development teams, where uncertainty, time pressure, and reduced social cues intensify both coordination demands and emotional strain. Drawing on social exchange theory and leader–member exchange theory, this study investigates how e-leadership influences team performance through team emotional capability and team engagement.

**Methods:**

A mixed-method design was used. Preliminary qualitative work, including literature review and focus group discussions, informed the measurement of e-leadership in distributed software development teams. Survey data from distributed new product development teams in Türkiye were analyzed using partial least squares structural equation modeling with SmartPLS.

**Results:**

E-leadership did not have a significant direct effect on team performance. However, it had a significant indirect effect through team emotional capability, indicating an indirect-only mediation pattern. Team emotional capability also mediated the relationship between e-leadership and team engagement. The sequential mediation pathway from e-leadership to team performance through team emotional capability and team engagement was not supported.

**Discussion:**

The findings show that e-leadership improves distributed team outcomes primarily by strengthening collective emotional capability rather than by directly increasing performance. Team emotional capability appears to be a critical affective mechanism through which digitally mediated leadership supports engagement and performance in distributed new product development teams. The study contributes to e-leadership research by clarifying the emotional pathway linking digital leadership practices to team effectiveness and offers practical guidance for leaders seeking to build resilient, engaged, and high-performing distributed teams.

## 1 Introduction

The rapid proliferation of distributed work, characterized by geographically dispersed teams, has fundamentally transformed organizational collaboration and redefined leadership paradigms in the digital era. Enabled by digital technologies, distributed teams transcend physical boundaries, allowing individuals to collaborate across diverse locations and time zones (Bosch-Sijtsema et al., 2011; Stubbs and Baghurst, 2014). While this model offers unparalleled flexibility and access to global talent, it also introduces significant challenges stemming from geographical, cultural, and organizational diversity. Addressing these complexities requires leadership approaches that move beyond traditional competencies, emphasizing trust-building, seamless communication, and coordination in environments devoid of physical proximity (Hoch and Kozlowski, 2014).

Amid these shifts, e-leadership has emerged as a critical framework for navigating the intricacies of distributed work. E-leadership refers to the practices and strategies that leaders employ to overcome barriers associated with physical distance, technology-mediated communication, and virtual collaboration dynamics (Hinds and Bailey, 2003). Unlike conventional leadership, e-leadership demands a unique blend of technical expertise and the ability to inspire, motivate, and connect individuals within virtual environments. By effectively utilizing digital tools, e-leaders foster meaningful relationships, enhance team dynamics, and address the distinct challenges of distributed collaboration, ultimately leading to higher team performance (Gibbs et al., 2017).

The significance of e-leadership becomes particularly evident in distributed new product development (NPD) teams, where leaders must coordinate complex, knowledge-intensive work across geographical boundaries without the benefit of shared physical spaces. Unlike many routine e-teams, NPD teams operate under high uncertainty, iterative experimentation, and strong temporal pressures tied to release milestones and speed-to-market, while integrating diverse expertise to transform ambiguous requirements into deliverable product features (Eppinger and Chitkara, 2009). These characteristics amplify coordination demands and intensify the emotional load of collaborative work, because setbacks, rework, and shifting customer expectations are frequent and consequential in the innovation cycle (Badrinarayanan and Arnett, 2008). Traditional mechanisms for fostering team cohesion and emotional connections are often inadequate in these contexts, necessitating tailored strategies and innovative approaches to build high-performing teams (Driskell et al., 2003; Berry, 2011). By leveraging digital tools and adopting people-focused strategies, e-leaders not only mitigate the limitations of virtual collaboration but also enhance the collective capacity of teams to innovate and adapt (Daswin et al., 2022; Kiljunen et al., 2021). In this context, team performance is intrinsically linked to a leader’s ability to balance task-related responsibilities and interpersonal dynamics, underscoring the pivotal role of leadership in achieving desired outcomes (Hill and Bartol, 2016; Wang et al., 2014; Shuffler et al., 2010).

In overcoming the barriers inherent in distributed settings, team emotional capability, a team’s collective ability to recognize, understand, and manage emotions, emerges as a pivotal factor. Physical separation often undermines emotional bonding, yet emotional intelligence plays a critical role in promoting collaboration and strengthening team dynamics. By regulating personal and interpersonal emotions, teams can enhance their resilience and functionality (Cole et al., 2016). Evidence further suggests that interventions targeting emotional management effectively address the deficits of virtual environments, bolstering team cohesion and significantly improving performance in NPD projects (Holtz et al., 2020; Akgün et al., 2011).

Complementary to emotional capability, team engagement, defined as a shared sense of purpose, energy, and commitment toward organizational goals, also holds a central role. Emotionally intelligent behaviors have been shown to enhance engagement by fostering stronger interpersonal connections within teams (Bakker, 2022; Damasceno et al., 2021). E-leaders play a vital role in cultivating engagement by building trust, enabling open communication, and aligning team efforts with organizational objectives (Vesala-Varttala and Morgado, 2022). The interplay between emotional capability and engagement forms the foundation for addressing the unique demands of distributed teams while maximizing their overall performance.

Drawing on social exchange theory (SET) and leader–member exchange (LMX) theory, we conceptualize e-leadership as an ICT-mediated influence process that shapes reciprocal, trust-based exchanges in distributed work. SET explains why leaders’ digitally enacted support, responsiveness, and transparency provide socioemotional resources that team members feel obligated to reciprocate through greater collective effort, information sharing, and coordination (Blau, 1964; Cropanzano and Mitchell, 2005). LMX complements this logic by emphasizing that reciprocity is built through repeated leader–member interactions; in distributed settings, where interactions are largely technology-mediated and cues are reduced, the consistency and quality of these micro-exchanges become more difficult to establish and therefore more consequential for team functioning (Graen and Uhl-Bien, 1995; Van Wart et al., 2019a, 2019b). Accordingly, we theorize that e-leadership influences team performance primarily through team emotional capability, a collective affective resource enabling members to recognize, regulate, and leverage emotions for coordination and learning, and through team engagement as a shared motivational state.

Despite notable advances, several important gaps remain unaddressed in the current literature. First, e-leadership is frequently conflated with adjacent constructs (e.g., transformational, digital leadership), which blurs conceptual boundaries and constrains cumulative knowledge; recent reviews and mappings continue to call for sharper demarcation and theory-building specific to ICT-mediated leadership (Tigre et al., 2023; Banks et al., 2022). Second, the causal pathways through which ICT affordances translate into performance are often under-specified, particularly the mediated mechanisms operating through team emotional capability and engagement that plausibly link leader behaviors to collective outcomes in distributed teams (Alkhayyal and Bajaba, 2024; Bieńkowska et al., 2023). Third, mixed-methods designs seldom document with procedural transparency how qualitative insights inform survey instrumentation (e.g., item generation/adaptation), limiting interpretability and replication; at the same time, performance tends to be operationalized fragmentedly, with time-to-market and quality rarely modeled jointly within a single explanatory framework (Contreras et al., 2020; Banks et al., 2022). Fourth, contextual boundary conditions, including task interdependence, team size/maturity, and project type, are under-examined as moderators, leaving open questions about scope and generalizability across technology-intensive settings (Banks et al., 2022). Finally, a nontrivial share of findings continues to rely on single-source, cross-sectional self-report data, which heightens vulnerability to common-method bias and obscures temporal dynamics critical to virtual collaboration (Alkhayyal and Bajaba, 2023; Banks et al., 2022). Collectively, these limitations underscore the need for clearer construct demarcation, mechanism-focused models that incorporate emotional processes, transparent mixed-methods chains, and more holistic performance indicators in future e-leadership research.

This study offers a dual contribution. Theoretically, it enriches the discourse by integrating emotional capability and engagement as mediating constructs in the e-leadership-performance relationship, contextualized through SET and LMX frameworks. Practically, it provides actionable strategies for leaders to enhance trust, cohesion, and productivity in distributed teams, particularly in specialized and technology-intensive environments.

Accordingly, this study seeks to address the following research questions:

1. How does e-leadership influence team performance in distributed settings?
2. To what extent do team emotional capability and engagement mediate the relationship between e-leadership and team performance?

To address these questions, the present study focuses on distributed software development teams operating in İstanbul and Kocaeli’s technology development zones. By examining the dynamics of e-leadership, emotional capability, and engagement, the research aims to provide actionable insights and theoretical contributions to the discourse on leadership in distributed work environments.

## 2 Literature review and hypotheses development

### 2.1 E-leadership: the evolving role of leadership in the digital era

For clarity, throughout this study we use the term virtual (distributed) team to refer to an interdependent group whose members work across spatial and/or temporal boundaries and rely primarily on digital communication technologies to coordinate their tasks and sustain collaboration (Gibson and Gibbs, 2006; Gilson et al., 2015). This definition is particularly salient for distributed NPD work, where interdependence is high and coordination is routinely enacted through ICT rather than co-presence.

We distinguish e-leadership from digital leadership to sharpen construct boundaries in a digitized work landscape. Digital leadership typically concerns steering digital transformation and technology-driven strategic change at the organizational level, whereas e-leadership focuses on the day-to-day social influence processes enacted through ICT to coordinate, motivate, and support dispersed collaborators (Avolio et al., 2014; Cortellazzo et al., 2019; Van Wart et al., 2019a, 2019b). Given our focus on distributed NPD teams and ICT-mediated collaboration routines, e-leadership offers the most precise conceptual anchor because it targets micro-level coordination and influence under media constraints.

E-leadership captures leadership as a technology-mediated social influence process through which leaders shape shared understanding, affective tone, and coordinated action in virtual environments (Avolio et al., 2014; Cortellazzo et al., 2019). Unlike traditional leadership, which relies on direct, co-present interaction, e-leadership is shaped by digitally mediated tools and platforms and therefore hinges on deliberate choices about media, visibility, and interaction routines (Avolio et al., 2014; Van Wart et al., 2019a, 2019b). Theoretically, its lineage cuts across organizational behavior, information systems, and leadership studies: early accounts emphasized in-person influence (Bass, 1985), but the rise of virtual teams forced a re-examination of leadership paradigms to address the complex, technology-mediated realities of remote collaboration (Zaccaro and Bader, 2003). Transformational and distributed leadership contributed vital building blocks, articulating vision and elevating collective motivation (Bass, 1990; Avolio et al., 2014) while enabling shared decision-making via platforms that connect geographically dispersed contributors (Yammarino et al., 2015). Yet e-leadership foregrounds the “how” of influence in distributed work—how coordination, motivation, and support are engineered when interaction is primarily ICT-based—making it directly relevant to the operating realities of distributed NPD teams. As leadership becomes enacted through ICT, the same technologies that enable speed and reach also introduce new coordination and relational frictions.

This digital turn creates opportunities and friction. Leaders must foster adaptability and innovation while navigating resistance to change (Cortellazzo et al., 2019). Physical distance disrupts conventional supervision and cohesion mechanisms; therefore, trust must be engineered through transparency in decision-making, cadence-based digital check-ins, and psychologically safe norms in virtual teams (Jawadi et al., 2013). Related constructs, digital leadership and cyber leadership, illuminate contiguous territories (Cortellazzo et al., 2019; Malakyan, 2019), yet e-leadership positions technology not merely as backdrop but as the primary enabler of strategy execution, communication, and collaboration at scale. As organizations digitize, leaders must master technology-mediated communication, asynchronous coordination, and motivational design without the benefit of co-presence (Iriqat and Khalaf, 2017; Hoch and Kozlowski, 2014). Empirically, e-leadership enhances performance by cultivating cohesive team dynamics via platform-enabled support and clarity (Sanchez et al., 2023) and by deploying electronically mediated channels, from email and video conferencing to enterprise social networks, competently and intentionally (Van Wart et al., 2019a, 2019b). While SET clarifies reciprocity, obligation, and trust as the social underpinnings of leadership, digitally mediated work redefines how those exchanges are enacted, through media choices, visibility architectures, and explicit coordination rules that substitute for the missing cues of co-presence. Recent research continues to underscore the centrality of leadership-enabled trust and coordination in virtual teams and digital work arrangements (Abakpa and Dvouletý, 2025; Kloepfer and Carbon, 2025; Chen et al., 2026).

Against this backdrop, conceptual demarcation becomes essential to avoid conflating e-leadership with adjacent models largely theorized for co-present, synchronous settings. Transformational leadership presupposes high-richness exchanges, frequent face-to-face contact, dense emotional cues, and shared temporal rhythms that allow inspirational framing and individualized consideration to propagate effectively; in geographically dispersed teams, media-reduced channels attenuate such cues and raise the transaction costs of sense-giving and sense-making, so behaviors that depend on collocated resonance often yield diminishing returns unless redesigned for mediated contexts (Greimel et al., 2023). Agile leadership excels at adaptive iteration, yet canonical practices (daily stand-ups, osmotic communication, co-located boards) assume tight temporal coupling and stable ceremonies; distributed programs operate under asynchronous constraints, time-zone fragmentation, and fluctuating participation windows, and without explicit re-engineering these routines can become coordination-heavy rather than flow-enabling (Iannotta et al., 2020; Coun et al., 2021). Digital leadership foregrounds strategic technology orientation, vision, investment, and culture for digitization, yet often remains macro-level in focus, offering less guidance on the micro-coordination by which tools translate into day-to-day collaboration efficacy; visionary roadmaps are necessary but insufficient without clear specifications for tool-stacks, protocols, and cadences that minimize handoff frictions and information asymmetries in distributed projects (Cortellazzo et al., 2019; Fatima and Masood, 2023).

By contrast, e-leadership is defined by the orchestration of ICT affordances to jointly optimize information visibility and workflow coupling (persistent channels, shared backlogs, issue trackers), communication cadence and media fit (rules for when to go async vs. sync; “written-first, meeting-last”), and emotional scaffolding in low-cue environments (ritualized recognition, expectation clarity, psychological safety). This orchestrational stance reframes leadership as coordination design under media constraints: selecting and sequencing channels, defining lightweight decision rights and escalation paths, and engineering trust when proximity is absent, thereby addressing the specific failure points of adjacent approaches (asynchronicity, cue scarcity, coordination cost) and providing a mechanism-ready bridge to outcomes via technology-enabled collaboration, team emotional capability, and engagement (Bauwens and Cortellazzo, 2025; Juknevičienė et al., 2025).

Practically, effective e-leaders translate strategy into visible operating decisions and responsibilities: they make coordination explicit (who updates what, where, and by when?), select media on purpose (async by default; sync for ambiguity or conflict), and treat latency and missing cues as design constraints rather than excuses, establishing predictable cadences that stabilize attention, reduce switching costs, and sustain motivation. The result is leadership through the intelligent architecture of interaction, where exchanges are reliable, obligations are transparent, and performance emerges as the by-product of a well-designed digital workplace (Cortellazzo et al., 2019; Avolio et al., 2014; Van Wart et al., 2019a, 2019b; Hoch and Kozlowski, 2014).

### 2.2 The impact of e-leadership on team performance in distributed work environments

As distributed work settings in general, distributed team settings in particular, increasingly replaces traditional ones, team members often experience challenges such as reduced compliance, weakened trust, interpersonal conflicts, fragmented knowledge sharing, and lower team cohesion (Reyes et al., 2021; MacDuffie, 2007). Consequently, the role of e-leaders in shaping team effectiveness and driving performance has become increasingly critical.

From a theoretical standpoint, social exchange theory (Blau, 1964) and leader–member exchange (LMX) theory (Graen and Uhl-Bien, 1995) help explain how e-leadership translates into performance in distributed NPD settings. Through digitally enacted support, fairness, and timely communication, e-leaders provide socioemotional resources and reduce coordination uncertainty; these cues invite reciprocity in the form of greater collective effort, mutual helping, and information sharing, which should enhance performance (Cropanzano and Mitchell, 2005). Consistent with LMX logic, repeated high-quality leader–member interactions also shape how members interpret leader intent and develop trust in technology-mediated collaboration, thereby influencing shared team states (e.g., emotional capability and engagement) that are proximal drivers of NPD performance.

Beyond interpersonal relationship mechanisms, e-leadership strategically enhances key team processes that drive performance, including team cohesion, communication quality, and conflict management (Han et al., 2022; Powell et al., 2004). Scholars (e.g., Islami, 2020) suggest that open communication and team cohesion are critical determinants of performance, particularly in virtual settings where shared mental models and collective coordination become paramount. Zhang et al. (2021) further highlight that authentic leadership fosters interpersonal trust and cohesion in virtual teams, ultimately strengthening team performance.

Moreover, the role of shared leadership, where leadership responsibilities are distributed across team members, has been recognized as a key factor in virtual team effectiveness (Ali and Jiang, 2025). Klasmeier and Rowold (2021) argue that transformational leadership, a fundamental aspect of e-leadership, cultivates shared leadership structures, which in turn facilitate greater team success. Fausing et al. (2015) and Carte et al. (2006) emphasize that empowering leadership behaviors enhance shared leadership, thereby improving performance outcomes. This perspective aligns with findings that shared leadership is particularly beneficial in complex task environments, as it fosters greater adaptability and problem-solving capacity (Hadi, 2021).

Given the strategic role of e-leadership in structuring team interactions, fostering high-quality exchanges, and enabling shared leadership, it is reasonable to expect that e-leadership has a direct and significant impact on team performance in distributed teams. Accordingly, we propose the following hypothesis:

*H1:* E-leadership has a significant positive effect on team performance in distributed teams.

#### 2.2.1 Team emotional capability as a mediator in the e-leadership–performance relationship

E-leadership is particularly relevant in enhancing team emotional capability, which refers to a team’s collective ability to perceive, interpret, regulate, and leverage emotions to foster a productive and cohesive work environment (Ghuman, 2011; Cole et al., 2016). Given the inherent challenges of remote interactions, where non-verbal cues are limited, the ability of e-leaders to foster emotional awareness and trust through digital communication becomes essential. Effective e-leadership not only supports the recognition of team members’ emotional states but also strengthens the emotional flow within the team, ultimately contributing to higher levels of engagement, collaboration, and psychological safety (Holtz et al., 2020; Eslamdoust et al., 2024).

Moreover, the capacity of distributed teams to maintain high emotional capability has been linked to improved team performance. Emotional capability enables teams to manage the affective dimensions of collaboration, influencing key aspects such as decision-making, engagement, and problem-solving (Kayworth and Leidner, 2001; Gibson and Gibbs, 2006). In distributed settings, where physical separation may hinder spontaneous social interactions, e-leaders play a pivotal role in fostering emotional bonds and facilitating shared emotional experiences that enhance team cohesion and collective efficacy (Jordan and Troth, 2004). This aligns with evidence suggesting that emotional capability contributes to higher adaptability, conflict resolution, and overall performance outcomes (Côté and Miners, 2006; Farh et al., 2012). This need is especially pronounced in NPD project teams, where emotional capability supports learning from iteration and helps sustain coordination under deadline pressure (Akgün et al., 2011).

Furthermore, some research has increasingly emphasized the mediating role of team emotional capability in the relationship between leadership and performance outcomes. Studies indicate that emotionally competent teams are better equipped to manage trust, collaboration, and stress, leading to improved project success rates (Akgün et al., 2009; Frye et al., 2006). Specifically, Akgün et al. (2011) provide empirical evidence that team emotional capability enhances project outcomes by facilitating knowledge integration and emotional synergy. Similarly, Rezvani et al. (2018) demonstrate that team emotional intelligence mediates the relationship between trust and performance, further reinforcing the argument that emotional capability is integral to team effectiveness.

Given these findings, emotional capability emerges as a crucial variable in understanding the relationship between e-leadership and distributed team performance. In light of this, we propose the following hypothesis to examine the role of emotional capability in mediating this relationship:

*H2:* The relationship between e-leadership and team performance in distributed teams is mediated by team emotional capability.

#### 2.2.2 Team emotional capability as a mediator in the e-leadership–team engagement relationship

E-leadership plays a pivotal role in shaping team emotional capability, which refers to a team’s ability to recognize, regulate, and leverage emotional dynamics to facilitate collaboration and performance (Cole et al., 2016). Through effective leadership practices, e-leaders influence key team dynamics, including engagement, communication, trust, and cohesion (Kashive et al., 2022; Kayworth and Leidner, 2001; Zigurs, 2003). One of these critical dynamics, team engagement, is characterized by the physical, emotional, and cognitive investment of team members in their work (Kahn, 1990; Saks, 2006). Scholars (e.g., Brosi and Schuth, 2020), in this meaning, suggest that team engagement is shaped not only by individual motivation but also by the leadership style and the emotional Kahsive climate within the team.

The ability of e-leaders to create a supportive and psychologically safe environment is fundamental to sustaining high engagement levels in distributed teams. Leaders who effectively utilize digital communication channels to provide motivation, trust, and constructive feedback can enhance employee engagement, particularly in virtual work environments where spontaneous interactions are limited (Panteli and Yalabik, 2019). Hassan (2024) emphasizes that e-leadership, by leveraging digital technologies, fosters greater engagement among team members by facilitating transparent communication and emotional support. Additionally, research highlights that teams with strong emotional capability, enabled by e-leadership, are more adept at managing conflict, fostering collaboration, and maintaining motivation, all of which contribute to heightened engagement (Mayer et al., 2008).

Moreover, the integration of technological tools in leadership interactions, commonly referred to as e-leadership, has been found to enhance leader-member exchange (LMX) quality, thereby reinforcing trust, job satisfaction, and performance outcomes (Eslamdoust et al., 2024). Leaders with high emotional intelligence can navigate the affective landscape of their teams, leading to greater engagement and cohesion (Hur et al., 2011; Waglay et al., 2020). Consequently, team emotional capability emerges as a key mediating factor in the relationship between e-leadership and team engagement (Koutsioumpa, 2023; Aritzeta et al., 2020). This argument is further supported by Lee et al. (2021), who found that ethical leadership mitigates emotional exhaustion, preserving employees’ emotional resources and fostering engagement. Similarly, Huang (2022) identified emotion regulation as a mediating mechanism between leadership styles and work engagement, highlighting the importance of leaders’ ability to manage both their own emotions and those of their team to sustain high engagement levels.

The intricate relationship between e-leadership, team engagement, and team emotional capability underscores the need for leaders to cultivate an environment that promotes engagement and psychological well-being (Carasco-Saul et al., 2014; Mazzetti, 2022). Furthermore, e-leaders can enhance team performance by strengthening emotional capability, which, in turn, influences engagement, communication, decision-making, collaboration, and trust (Eslamdoust et al., 2024; Kashive et al., 2022). As organizations continue to refine remote work strategies, developing a comprehensive understanding of these interdependencies is essential for building resilient, high-performing virtual teams (Sutisna et al., 2024).

When examining the relationship between e-leadership, team engagement, and team emotional capability, initial observations suggest that both engagement and emotional capability are influenced by e-leadership. However, a deeper analysis is required to determine whether team emotional capability mediates the relationship between e-leadership and team engagement. Accordingly, we propose the following hypothesis:

*H3:* The relationship between e-leadership and team engagement in distributed teams is mediated by team emotional capability.

#### 2.2.3 The interplay of e-leadership, team performance, emotional capability, and engagement in distributed teams

The impact of e-leadership on team performance is neither linear nor immediate; rather, it unfolds through intermediary mechanisms that address the psychosocial complexities of virtual collaboration. Team emotional capability, the collective ability to perceive, regulate, and leverage emotions effectively, plays a crucial role in facilitating positive interpersonal dynamics (Van Kleef et al., 2009; Rezvani et al., 2018). Additionally, team engagement, defined as the shared sense of psychological involvement, motivation, and commitment, serves as a key driver of performance outcomes (Schaufeli et al., 2009; Contreras et al., 2020). These two mechanisms operate in a sequential manner, whereby e-leadership first enhances team emotional capability, which subsequently fosters team engagement, ultimately leading to superior team performance.

E-leadership is not merely about digital coordination; it is about shaping the team’s emotional and psychological environment in ways that compensate for the absence of physical proximity (Hoch, 2014). The ability to model emotional intelligence behaviors, create a psychologically safe space, and establish norms for open emotional expression is crucial for e-leaders in virtual settings (Van Kleef et al., 2009). In remote environments, where social cues are diminished, e-leaders must proactively cultivate emotional intelligence within teams to maintain cohesion and resilience (Golden and Veiga, 2008). Teams with strong emotional capability are better equipped to navigate interpersonal conflicts, sustain morale, and regulate stress, factors that are particularly vital yet often overlooked in distributed teams (Boyatzis et al., 2013; Akgün et al., 2011). The absence of physical interactions makes these teams more susceptible to miscommunication, emotional detachment, and decreased trust, all of which can erode team effectiveness (Gibson and Gibbs, 2006; Kashive et al., 2022). E-leaders mitigate these risks by fostering an emotionally intelligent team culture, wherein members feel heard, valued, and psychologically secure (Açıkgöz et al., 2013; Contreras et al., 2020).

Beyond conflict resolution, emotional capability also energizes team members by promoting positive emotional contagion (Fortuin et al., 2023). Bakker (2022) suggests that emotionally intelligent teams create a reinforcing cycle, where shared positive emotions enhance engagement, which in turn strengthens team collaboration and performance. Thus, team emotional capability acts as the first mechanism through which e-leadership translates into higher team performance.

While emotional capability lays the foundation for effective team interactions, it must be activated and sustained through engagement, the degree to which team members are psychologically invested, motivated, and actively contributing to team objectives (Schaufeli et al., 2009). Engaged teams exhibit higher creativity, problem-solving ability, and resilience, all of which are critical for success in virtual environments (Contreras et al., 2020). Empirical evidence suggests that teams with strong emotional capability are more likely to be engaged, as they experience less interpersonal tension, greater social support, and a shared sense of purpose (Bakker, 2022; Patel et al., 2021). When teams are emotionally capable, members feel more connected, valued, and committed to their collective goals (Gibson and Gibbs, 2006). This sense of belonging amplifies engagement, fostering a proactive work ethic and intrinsic motivation (Salanova et al., 2005).

Engagement serves as the ultimate pathway through which team emotional capability translates into enhanced performance. Research consistently shows that engaged teams demonstrate higher efficiency, adaptability, and overall output, particularly in remote work settings where autonomy and self-regulation are key determinants of success (Schaufeli et al., 2009; Contreras et al., 2020). Moreover, engaged teams experience fewer coordination breakdowns, as members are more inclined to actively participate, take initiative, and sustain focus despite the lack of physical presence (Gilson et al., 2015). The reciprocal relationship between team cohesion and performance further reinforces this dynamic, teams that become more cohesive due to effective leadership and emotional regulation subsequently improve their performance, which in turn strengthens engagement and collaboration (Patel et al., 2021; Mathieu et al., 2015). This cyclical effect underscores the strategic imperative for e-leaders to prioritize both emotional and engagement-based interventions to drive sustainable team effectiveness (Robert, 2013). Hence, we propose the following sequential mediation hypothesis:

*H4:* The relationship between e-leadership and team performance in distributed teams is mediated by team emotional capability and team engagement, respectively.

**Figure 1:**
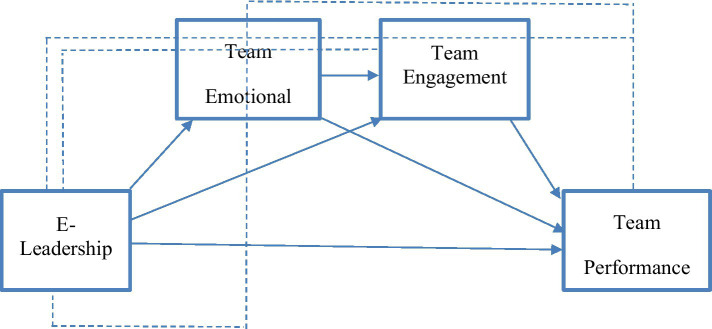
Research model. ----- denotes the indirect relationships.

In line with the hypotheses discussed above, the conceptual model for our study has been developed as follows. This model illustrates both direct and indirect relationships (Figure 1).

## 3 Methodology

This study investigates the interrelationships between e-leadership, emotional capability, team engagement, and team performance within the context of distributed New Product Development (NPD) teams. To achieve the research objectives, a two-step methodological approach was adopted, combining qualitative and quantitative methods. First, preliminary qualitative investigations were conducted through an extensive literature review and focus group discussions. Second, a survey was administered to software development teams to generate conclusive quantitative insights. This mixed-method design was selected to ensure comprehensive, robust, and externally valid findings (Davis et al., 2011). E-leadership, as defined by Roman et al. (2019), encompasses a set of technology-mediated social influencing processes. However, the competencies required for effective e-leadership remain underdeveloped and lack a standardized definition or measurement framework. This gap in the literature is further complicated by inconsistencies across studies in defining and measuring e-leadership qualities (Subramaniam, 2023). Frich et al. (2015) highlight significant variability in leadership development programs, illustrating the broader challenge of constructing a universally accepted framework for e-leadership.

Given these ambiguities, the study employed an exploratory approach to disentangle the critical facets of e-leadership in software development contexts. Two focus groups were conducted with distributed software professionals (*n* = 8; 3 team leaders, 5 team members) selected purposefully for role, skill, and experience fit. The group size and composition are consistent with recommendations for non-commercial focus groups that balance depth with manageable interaction dynamics (Krueger, 2000; Krueger and Casey, 2014; McLure et al., 2014). Each ~90-min discussion was audio-recorded and transcribed; open-ended, discovery-oriented questions solicited rich qualitative data (e.g., “How would you describe effective leadership in distributed software development teams?”; “What are the components of effective leadership in remote or hybrid team settings?”).

The data analysis process employed open coding supported by NVivo. Participant statements were segmented into meaning units and coded, then grouped under similar themes to form overarching categories. We examined code frequencies and interrelations between themes to identify critical elements. Focus-group themes directly informed item generation and scale selection. For each theme, we extracted exemplar verbatims, articulated observable behavioral rules for distributed work (e.g., async-first cadence), and then either wrote new items or adapted well-cited items to the ICT-mediated context. Every item’s provenance (New/Adapted/Adopted), wording changes, and theme link is documented in Table 1 (Item Provenance & Wording Changes). Items underwent editorial refinements (clarity, specificity, brevity) based on expert review and brief cognitive checks; changes are listed in the “Refinement (editorial)” column. Inter-coder reliability was assessed via independent double-coding by two researchers and reached 85% agreement, ensuring robustness and reliability. The focus-group insights emphasized a dual capability profile for e-leaders, e-communication skills and digital literacy, and described proficiency in facilitating technology-mediated communication, addressing the social needs of geographically dispersed members, and fostering sociability in virtual environments. Practices such as daily meetings with high participation, online team-building activities, and consistent use of digital tools were highlighted as essential. These insights underscore the necessity for e-leaders to integrate technical proficiency with interpersonal effectiveness to cultivate cohesive and high-performing teams in digital contexts.

**Table 1:** Qual → Quant audit trail: focus-group themes mapped to survey items.

Theme (from focus groups)	Exemplar verbatim (anonymized)	Item wording (survey; retained)	Source	Refinement (editorial)
Visibility & cadence	If it is not in the tracker, it does not exist.	Our leader requires updates to reside in shared trackers before meetings.	New (qualitatively derived)	Added “before meetings” for specificity
Async-first media rule	We default to async; we meet only when clarity is low.	We default to asynchronous channels and switch to meetings only when ambiguity remains.	Adapted (cf. Hoch and Kozlowski, 2014)	Replaced “clarity is low” with “ambiguity remains”
Clarity & responsiveness norms	Write it clearly and respond within the agreed window.	Written communication is expected to be concise and timely in line with team norms.	Adapted (cf. Van Wart et al., 2019a, 2019b)	Added “in line with team norms”
High-participation daily meetings	Daily check-ins keep everyone aligned.	Short daily check-ins ensure broad participation and shared situational awareness.	New (qualitatively derived)	Specified “short” to avoid meeting overload
Online team-building rituals	Virtual coffee/games help us feel like a team.	Regular online team-building activities strengthen our sense of belonging.	New (qualitatively derived)	—
Consistent collaboration platforms	Pick a tool and stick to it.	We consistently use the same collaboration platforms to coordinate work and decisions.	Adapted (cf. Van Wart et al., 2019a, 2019b)	Added “coordinate work and decisions”

Source indicates item provenance: New = qualitatively derived from focus-group themes; Adapted = modified from published items to fit distributed/ICT-mediated contexts; Adopted = taken verbatim from the literature. Adaptation references for the excerpted items: async/sync rule (cf. Hoch and Kozlowski, 2014) and e-communication/tool-use competency (cf. Van Wart et al., 2019a, 2019b). Items underwent editorial refinements (clarity, specificity, brevity) based on expert review and cognitive checks; wording changes are reported in the “Refinement (editorial)” column.

Participants also noted that, while e-leadership relies on digital tools, its fundamental principles align closely with traditional face-to-face leadership: effective e-leaders possess vision, role-model, support individual development, and inspire their teams, qualities resonant with transformational leadership. Roman et al. (2019) argue that e-leadership mirrors transformational leadership in leveraging ICT-mediated communication to build relationships and facilitate effective leadership. Similarly, Groves and LaRocca (2012) emphasize the transformational leader’s ability to instill purpose and motivate followers, a capability equally critical for e-leaders navigating digital transformation. Khan et al. (2018) further illustrate how transformational leaders drive organizational innovation, a quality vital for e-leaders adapting to dynamic digital environments. Supporting this perspective, Sukmawati et al. (2024) identify e-leadership as a paradigm shift that integrates technology to enhance communication and collaboration, and Hansbrough and Schyns (2018) highlight the role of shared vision and belonging, attributes equally relevant in e-leadership contexts. To measure e-leadership in a way that reflects these findings, the study operationalizes e-leadership by integrating two complementary scales: a four-item e-communication skills scale (Van Wart et al., 2019a, 2019b) and the team-level transformational leadership scale (Podsakoff et al., 1990); Turkish version validated by İşcan (2002). This combination reflects the dual capability profile identified in the focus groups, where e-leaders were consistently described as requiring both proficiency in technology-mediated communication and the ability to inspire, support, and guide their teams in ways resonant with transformational leadership. While digital literacy also emerged as an important dimension during the qualitative phase, it was excluded from the final instrument because it was considered a baseline competency among software professionals in the sample. The resulting composite measure thus captures e-leadership as the convergence of digital communication behaviors and transformational leadership practices, directly aligning with both theoretical foundations and empirical insights.

### 3.1 Measures

To test the hypotheses formulated in this study, multi-item scales from established research were adopted to measure the variables of interest. Each variable was assessed using a 5-point Likert scale, ranging from “strongly disagree” (1) to “strongly agree” (5). Detailed descriptions of the specific measures utilized in this study are provided in the appendix. The questionnaire was administered in Turkish (the working language of participants). Where validated Turkish versions existed (e.g., transformational leadership; İşcan, 2002), we used those items. For scales without an established Turkish validation, we applied a forward–back translation procedure with bilingual researchers and resolved discrepancies through consensus to ensure semantic equivalence, following established cross-cultural translation guidelines (Brislin, 1970; Brislin, 1980; Beaton et al., 2000). A small pilot with software professionals confirmed item clarity and contextual fit prior to full deployment.

The qualitative signal pointed to two facets of e-leadership that matter in distributed software teams: technology-mediated communication behaviors (visibility, cadence, media fit, clarity) and team-level transformational leadership (shared vision, recognition, belonging). Accordingly, we combined an e-communication item set (New/Adapted to distributed work) with a team-referent transformational leadership set (Adapted/Adopted). Digital literacy, although salient qualitatively, was omitted to avoid redundancy given baseline proficiency in our sampling frame. Item-to-theme traceability appears in Table 1.

To measure e-leadership, we used a four-item e-communication skills scale adopted from Van Wart et al. (2019a, 2019b), alongside Podsakoff et al.’s (1990) transformational leadership scale, whose Turkish version was developed and validated by İşcan (2002). Consequently, the final scale consists of 27 items across six dimensions, distributed as follows: 4 items for e-communication skills, 8 for vision-inspiring role model, 4 for fostering acceptance of group goals, 4 for intellectual stimulation, 4 for individualized support, and 3 for high-performance expectations. All items were structured on a 5-point Likert scale to gauge varying levels of agreement. Examples of scale items include: “My team leader actively engages with team members through online platforms to foster collaboration and facilitate knowledge sharing,” reflecting e-communication skills; “My team leader effectively inspires others to commit to and align with his/her vision,” representing the vision-inspiring role model dimension; and “My team leader motivates team members to collaborate and function as cohesive team players,” capturing fostering acceptance of group goals. Other examples are: “My team leader poses thought-provoking questions that stimulate critical thinking and innovation,” exemplifying intellectual stimulation; “My team leader consistently demonstrates respect and sensitivity toward my personal feelings and concerns,” illustrating individualized support; and “My team leader sets and communicates high performance expectations, encouraging us to strive for excellence,” reflecting high-performance expectations. These items are carefully designed to comprehensively capture the multifaceted nature of e-leadership.

To assess team emotional capability, we adopted the Team Emotional Capability Scale developed by Akgün et al. (2007) and Akgün et al. (2011). This scale evaluates six key dimensions: encouragement, displaying freedom, playfulness, experiencing, reconciliation, and identification. It comprises a total of 22 items, distributed as follows: 4 items for encouragement, 2 for displaying freedom, 3 for identification, and 5 each for reconciliation and experiencing. Like the previous scale, each item was rated on a 5-point Likert scale to capture varying degrees of agreement. Examples include: “Our team has the ability to instill hope among all its members,” which reflects encouragement; “In our team, people are encouraged to express their full range of emotions without fear of reprisal,” representing displaying freedom; “Our team creates a context that encourages experimentation,” highlighting playfulness; “People in our team experience the same or appropriate emotions in response to others’ feelings,” illustrating experiencing; “Our team can bring together two seemingly opposing values that people feel strongly about,” representing reconciliation; and “Members of our team express their deep attachment to salient team characteristics such as values and beliefs,” capturing identification. These carefully crafted items enable a holistic evaluation of team emotional capability.

To evaluate team engagement, the Team Engagement Scale developed by Costa et al. (2014) was utilized. This scale captures three core dimensions: vigor, absorption, and dedication, with 3 items allocated to each dimension, totaling 9 items. Example items include: “We are proud of the work that we do,” reflecting vigor; “We get carried away when we are working,” illustrating absorption; and “We are committed to achieving the team’s goals,” capturing dedication. These items are structured to provide a comprehensive understanding of the multifaceted nature of team engagement.

Lastly, to assess New Product Development (NPD) team performance, we combined the Product Quality Scale by Atuahene-Gima and Li (2006) with the Speed to Market Scale developed by Kessler and Chakrabarti (1999). The resulting scale evaluates team performance through two dimensions: product quality (5 items) and speed to market (3 items), with a total of 8 items. Examples of these items include: “The new product was perceived by customers as highly reliable compared to competing products,” representing product quality, and “This product (software) was launched on or ahead of the original schedule developed at the initial project go-ahead,” capturing speed to market. These measures collectively provide robust insights into project success, offering an integrated perspective on product quality, timeliness, and their broader organizational implications.

### 3.2 Sampling

The initial sample consisted of 150 firms specializing in software development, located in technology parks in Kocaeli and Istanbul. This selection was based on the strategic importance of these regions in Turkey’s technological ecosystem and their roles as hubs for innovation and entrepreneurship. Istanbul, as Turkey’s economic and technological epicenter, hosts a significant number of technology parks and innovation centers that facilitate collaboration between academia, industry, and government institutions. These facilities provide an ideal environment for software development teams, offering state-of-the-art infrastructure, access to funding, and proximity to a large talent pool (TÜBİTAK, 2023). Similarly, Kocaeli, with its industrial and technological orientation, has become a key region for technology-driven activities. Its proximity to Istanbul and its well-established technology parks, such as the TÜBİTAK Marmara Research Center and Bilişim Vadisi, make it a strategic choice for software development initiatives (KOSGEB, 2023).

The inclusion of teams from these regions ensured a robust and diverse sample, as they represent a variety of organizational sizes, project scopes, and work modalities. Additionally, software development is a core industry in these technology parks, contributing significantly to technological advancement and innovation in Turkey (Durak et al., 2022).

Data were collected between November 2024 and March 2025. To initiate data collection, the managers of the 150 firms were contacted by telephone, and the objectives of the study were explained. Of these, 103 firms agreed to participate. Within each participating software development project team, the team leader and at least two team members were invited to complete the survey. Team-member respondents were selected based on three criteria: direct involvement in the focal software development project, familiarity with the team’s day-to-day distributed collaboration processes, and sufficient project tenure to provide informed assessments of the team. Where more than two eligible team members were available, respondents were identified in consultation with the team leader and/or project coordinator to ensure that the information collected referred specifically to the focal project rather than to general organizational experiences.

To reduce single-source bias, data were collected from two independent respondent groups within each focal project team. Team members provided ratings of e-leadership, team emotional capability, and team engagement, whereas team leaders provided ratings of team performance. Importantly, team leaders evaluated the performance of the focal project team as a collective unit rather than evaluating individual team members. Specifically, leader-rated team performance captured the team’s new product development outcomes in terms of product quality and speed to market. This multi-source procedure was adopted to mitigate common method concerns and to avoid the conflation of perceptions across different projects.

To ensure respondent anonymity and encourage honest participation, participants were informed that their responses would remain confidential and would not be linked to them personally, to their firms, or to the software products developed. Questionnaires did not request personally identifying information, and responses were coded only at the team/project level for matching purposes. Individual team-member responses were aggregated to the team level prior to analysis. Team leaders did not have access to individual team-member questionnaires, and team members did not have access to the leader-rated performance questionnaire. Respondents were also informed that there were no right or wrong answers and were encouraged to answer as honestly as possible (Podsakoff et al., 2003).

**Figure 2:**
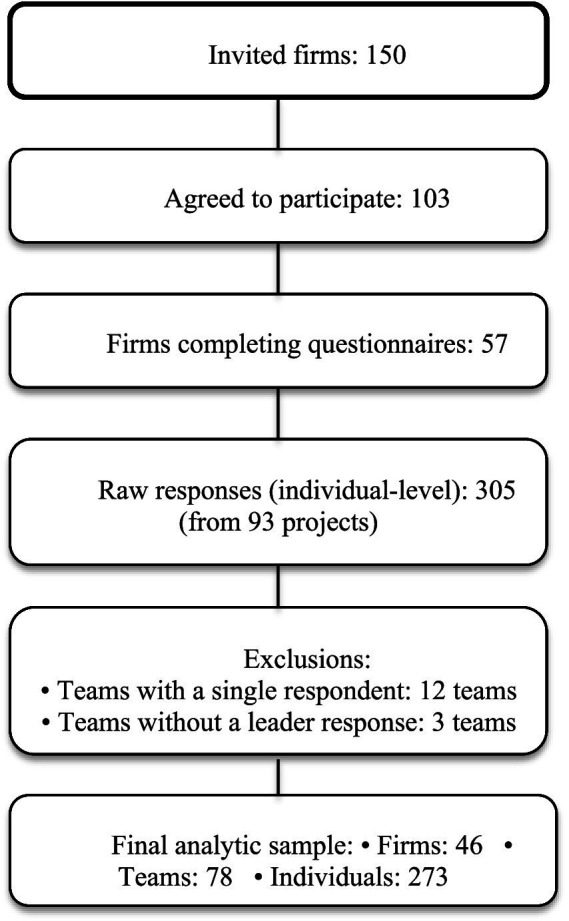
Sample flow diagram.

Of the 103 firms that agreed to participate, 57 firms completed the questionnaires, yielding 305 individual surveys from 93 software development projects. We applied a-priori team-level inclusion rules, requiring responses from the team leader and at least two team members. We excluded 12 teams with only one respondent and 3 teams without a leader response. The final analytic sample comprised 46 firms, 78 teams, and 273 individual surveys used in the main analyses (Figure 2). Because the structural model is estimated at the team level, the effective sample size is the number of teams (*N* = 78). This sample size is adequate for PLS-SEM given the model complexity, namely the maximum number of arrows pointing at an endogenous construct, and the expected medium effect sizes; we additionally verified statistical power using both a-priori and *post hoc* power assessments.

Table 2 presents a comprehensive summary of the demographic characteristics of the study’s participants, including gender, age, education, professional experience, team roles, team sizes, and work modalities.

**Table 2:** Demographics of the sample.

Variable	Category	*N*	(%)
Gender	Female	102	37.4
	Male	171	62.6
Age	23–27	85	31.1
	28–33	118	43.2
	34–47	70	25.6
Education level	High school/associate degree	18	6.6
	Bachelor’s degree	168	61.5
	Master’s/doctorate	87	31.9
Position level	Team leader	78	28.6
	Team member	195	71.4
Team size	3 members	27	34.6
	4 members	22	28.2
	5 members	17	21.8
	6 members	10	12.8
	More than 6 members	2	2.6
Work modality	Fully remote	128	46.9
	1 day in office	55	20.1
	2 days in office	82	30.0
	3 days in office	8	3.0
	4 days in office	0	0
	Fully office-based	0	0.0
Professional experience (leaders)	1–5 years	51	65.4
	6–10 years	18	23.1
	11 + years	9	11.5

The sample consists of 273 participants, including 102 females and 171 males, indicating a higher representation of male participants (62.6%). Participants’ ages range from 23 to 47 years, with an average age of 31.8 years (SD = 4.2). The majority of participants (43.2%) are aged between 28 and 33, while 31.1% are between 23 and 27, and 25.6% fall within the 34–47 age group. This distribution highlights a relatively young and dynamic workforce.

Educationally, the sample is highly qualified, with 168 participants (61.5%) holding a bachelor’s degree and 87 (31.9%) possessing a master’s or doctorate. Only a small minority (6.6%) have an associate degree or high school-level education, reflecting an advanced academic profile.

In terms of professional roles, the sample includes 78 team leaders (28.6%) and 195 team members (71.4%). Team sizes vary between 3 and 18 individuals, with smaller teams being more common. Specifically, 34.6% of teams consist of 3 members, 28.2% have 4 members, 21.8% have 5 members, and 12.8% have 6 members. Teams with more than 6 members are relatively rare, comprising only 2.6% of the sample.

The data also highlights diverse work modalities. Nearly half of the teams (46.9%) work fully remotely, while 20.1% attend the office one day per week. Most of the remaining participants (30.0%) adopt a hybrid model, working two days in the office, with only a small fraction (3.0%) attending the office three days a week. None of the teams are fully office-based, indicating the prevalence of flexible work arrangements in the sample.

Regarding professional experience, team leaders reported an average of 6.8 years of experience (SD = 2.7). Most leaders (65.4%) have 1–5 years of experience, followed by 23.1% with 6–10 years, and 11.5% with over 10 years. This distribution suggests that while some leaders possess extensive experience, the majority are relatively young in their careers.

Overall, the sample is characterized by a predominantly young and well-educated workforce, balanced representation of team leaders and members, and diverse team sizes and work modalities.

### 3.3 Analysis

The Partial Least Squares Structural Equation Modelling (PLS-SEM) technique was employed to estimate both the measurement and structural parameters within the structural equation model (SEM) (Chin, 2001). The selection of PLS-SEM for this study was informed by several methodological advantages.

First, PLS-SEM is particularly well-suited for models with a limited number of observations, as it effectively handles both discrete and continuous variables. As a latent variable modelling technique, it accommodates multiple dependent constructs while explicitly accounting for measurement error, ensuring robust results even in complex scenarios (Ringle et al., 2018).

Second, PLS-SEM offers significant advantages due to its less restrictive distributional assumptions. This makes it applicable in contexts where the distribution of latent variables is unknown. Unlike covariance-based structural equation modelling (CB-SEM), PLS-SEM aligns estimates more closely with empirical data, thereby enhancing model accuracy and reliability (Fornell and Cha, 1994).

Third, PLS-SEM demonstrates superior performance in managing intricate models, particularly those involving second-order variables with numerous sub-dimensions, such as those utilized in this study. While CB-SEM is generally recommended for simpler models with no more than eight latent variables, PLS-SEM excels in analysing more complex structures (Hair et al., 2016; Hair et al., 2019). These features establish PLS-SEM as a robust and appropriate method for investigating models incorporating latent variables and measurement errors.

Prior to analysis, the unit of analysis for this study was defined as the “software development team,” necessitating the aggregation of team-level scores for each question item. The inter-rater agreement (r_wg_) was calculated to ensure the reliability of these aggregated measures. The r_wg_ values ranged from 0.62 to 0.89, exceeding the established benchmark of 0.60 (Hurley and Hult, 1998). These results indicate a satisfactory level of inter-rater agreement, supporting the validity of the aggregated measures in the context of software development project teams (see Appendix).

### 3.4 Measurement validation

In this study, we employed reflective indicators for all constructs, following the approach of Kleijnen et al. (2007). To assess the psychometric properties of the measurement instruments, we initially estimated a null model that did not include any structural relationships. Internal consistency reliability was assessed through composite reliability (CR) and Cronbach’s alpha, whereas convergent validity was examined using the average variance extracted (AVE). After removing one item from product quality and another one from the dynamics of reconciliation due to its adverse impacts on the AVE, the constructs, e-leadership, team emotional capability, team engagement, and team performance, exhibited satisfactory reliability and validity. A detailed evaluation of the scales concluded that the removal of these two items did not compromise the content validity of their respective scales. The results indicated that the PLS-based CR values for all measures were well above the recommended threshold of 0.70. Similarly, Cronbach’s alpha values exceeded the threshold of 0.70, and the AVE values were either above or very close to the acceptable threshold of 0.50 (see Table 3). Convergent validity was further assessed by examining the standardized loadings of the items on their respective constructs. The findings demonstrated that all items exhibited standardized loadings greater than 0.60, indicating satisfactory convergent validity (see Appendix A).

**Table 3:** Validity and reliability.

Constructs	1	2	3	4	5	6	7	8	9	10	11	12	13	14	15	16	17
ABS	0.805																
DED	0.684	0.869															
DFR	0.448	0.485	0.924														
ENC	0.453	0.454	0.772	0.824													
EXP	0.380	0.500	0.543	0.580	0.855												
IDN	0.532	0.542	0.823	0.733	0.497	0.843											
PLY	0.578	0.464	0.643	0.682	0.388	0.700	0.806										
REC	0.640	0.784	0.585	0.612	0.582	0.625	0.471	0.787									
ECS	0.564	0.455	0.690	0.655	0.576	0.702	0.570	0.518	0.792								
GG	0.420	0.416	0.558	0.454	0.148	0.638	0.518	0.472	0.554	0.861							
HPE	0.386	0.432	0.355	0.355	0.609	0.293	0.207	0.465	0.444	0.084	0.863						
IS	0.536	0.598	0.652	0.631	0.502	0.637	0.541	0.688	0.554	0.625	0.455	0.868					
INS	0.394	0.483	0.510	0.472	0.324	0.611	0.403	0.529	0.624	0.591	0.181	0.595	0.810				
STM	-0.080	-0.003	0.125	0.117	0.006	0.042	-0.118	0.100	0.042	0.164	-0.161	0.057	0.161	0.879			
PQ	0.379	0.344	0.420	0.367	0.497	0.371	0.393	0.330	0.439	-0.019	0.528	0.274	0.159	0.029	0.762		
VIG	0.635	0.774	0.585	0.644	0.636	0.591	0.529	0.878	0.527	0.471	0.504	0.726	0.482	0.115	0.322	0.840	
VIS	0.390	0.462	0.653	0.601	0.336	0.681	0.542	0.609	0.531	0.622	0.290	0.638	0.580	0.059	0.145	0.622	0.803
α	0.740	0.837	0.830	0.836	0.907	0.805	0.733	0.842	0.806	0.913	0.832	0.836	0.825	0.856	0.761	0.790	0.921
CR	0.847	0.902	0.922	0.893	0.931	0.880	0.847	0.888	0.870	0.935	0.897	0.901	0.884	0.910	0.847	0.878	0.935
AVE	0.649	0.756	0.854	0.679	0.732	0.711	0.650	0.620	0.627	0.742	0.744	0.753	0.656	0.772	0.581	0.706	0.645

ABS: Absorption, DED: Dedication, DFR: Displaying Freedom, ENC: Encouragement, EXP: Experiencing, IDN: Identification, PLY: Playfulness, REC: Reconciliation, ECS: E-Communication Skills, GG: Fostering acceptance of group goals, HPE: High Performance Expectations, IS: Individual Support, INS: Intellectual Stimulation, STM: Speed to Market, PQ: Product Quality, VIG: Vigor, VIS: Vision-inspiring role model, α: Cronbach alfa, CR: Composite Reliability, AVE: Average Variance Extracted.

We next assessed the discriminant validity of the measures. Table 3 shows the correlation among all variables -excluding the secondary data on firm performance- that provide further evidence of discriminant validity. To fully satisfy the requirements for discriminant validity, AVE for each construct should be expected to be greater than the squared correlation between constructs (Fornell and Larcker, 1981). Such results suggest that the items share more common variance with their respective constructs than any variance the construct shares with other constructs (Howell and Avolio, 1993). In the model, none of the inter-correlations of the constructs exceeded the square root of the AVE of the constructs (see Table 3).

As an additional check for discriminant validity, we examined the heterotrait–monotrait (HTMT) ratio of correlations (Henseler et al., 2014). HTMT is a sensitive criterion in variance-based SEM and is assessed by comparing each construct pair’s HTMT value against recommended cutoffs (commonly < 0.90). As reported in Table 4, all HTMT values remain below 0.90 (highest = 0.897), providing additional support that the constructs are empirically distinct and that discriminant validity is established.

**Table 4:** HTMT results.

Constructs	1	2	3	4	5	6	7	8	9	10	11	12	13	14	15	16	17
ABS																	
DED	0.842																
DFR	0.560	0.581															
ENC	0.550	0.546	0.826														
EXP	0.430	0.572	0.633	0.689													
IDN	0.643	0.635	0.897	0.875	0.622												
PLY	0.762	0.588	0.813	0.843	0.463	0.874											
REC	0.763	0.860	0.720	0.746	0.659	0.764	0.602										
ECS	0.694	0.532	0.828	0.768	0.672	0.840	0.674	0.647									
GG	0.504	0.470	0.636	0.509	0.184	0.689	0.622	0.560	0.615								
HPE	0.454	0.515	0.421	0.437	0.674	0.358	0.268	0.552	0.530	0.112							
IS	0.652	0.717	0.780	0.751	0.576	0.745	0.689	0.825	0.651	0.715	0.552						
INS	0.485	0.580	0.613	0.561	0.376	0.723	0.503	0.686	0.761	0.677	0.214	0.712					
STM	0.116	0.137	0.167	0.162	0.114	0.169	0.152	0.182	0.100	0.199	0.190	0.147	0.205				
PQ	0.457	0.420	0.531	0.470	0.574	0.476	0.518	0.402	0.543	0.128	0.636	0.340	0.241	0.177			
VIG	0.782	0.850	0.722	0.797	0.748	0.740	0.688	0.763	0.639	0.556	0.616	0.894	0.595	0.214	0.393		
VIS	0.442	0.520	0.744	0.677	0.367	0.768	0.657	0.711	0.596	0.680	0.316	0.727	0.662	0.109	0.176	0.727	

ABS: Absorption, DED: Dedication, DFR: Displaying Freedom, ENC: Encouragement, EXP: Experiencing, IDN: Identification, PLY: Playfulness, REC: Reconciliation, ECS: E-Communication Skills, GG: Fostering Acceptance of Group Goals, HPE: High Performance Expectations, IS: Individual Support, INS: Intellectual Stimulation, STM: Speed to Market, PQ: Product Quality, VIG: Vigor, VIS: Vision.

**Figure 3:**
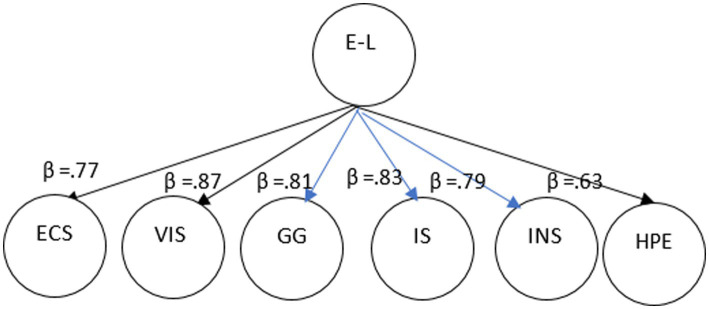
Second order factor analysis of e-leadership.

Moreover, e-leadership was modeled as a second-order construct comprising six first-order dimensions: e-communication skills (ECS), vision-inspiring role model (VIS), fostering acceptance of group goals (GG), intellectual stimulation (INS), individualized support (IS), and high-performance expectations (HPE). As shown in Figure 3, the standardized loadings of these six dimensions on the higher-order e-leadership construct exceeded the recommended threshold of 0.60. This result indicates that e-leadership, as a second-order construct, is significantly represented by ECS, VIS, GG, INS, IS, and HPE.

**Figure 4:**
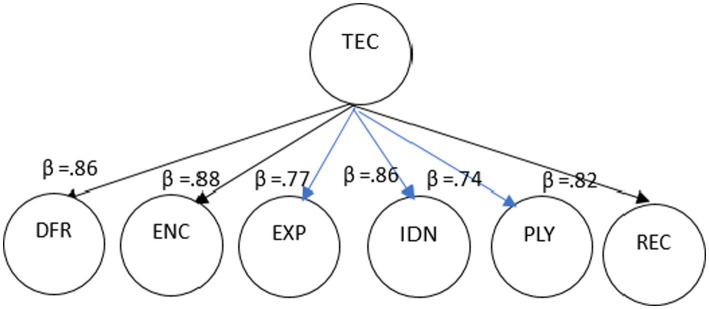
Second order factor analysis of team emotional capability.

Next, team emotional capability (TEC) was also modeled as a second-order construct comprising six first-order dimensions: encouragement (ENC), displaying freedom (DFR), playfulness (PLY), experiencing (EXP), reconciliation (REC), and identification (IDN). As shown in Figure 4, the standardized loadings of these six dimensions on TEC exceeded 0.60. This result suggests that TEC, as a six-dimensional second-order construct, is significantly represented by ENC, DFR, PLY, EXP, REC, and IDN.

**Figure 5:**
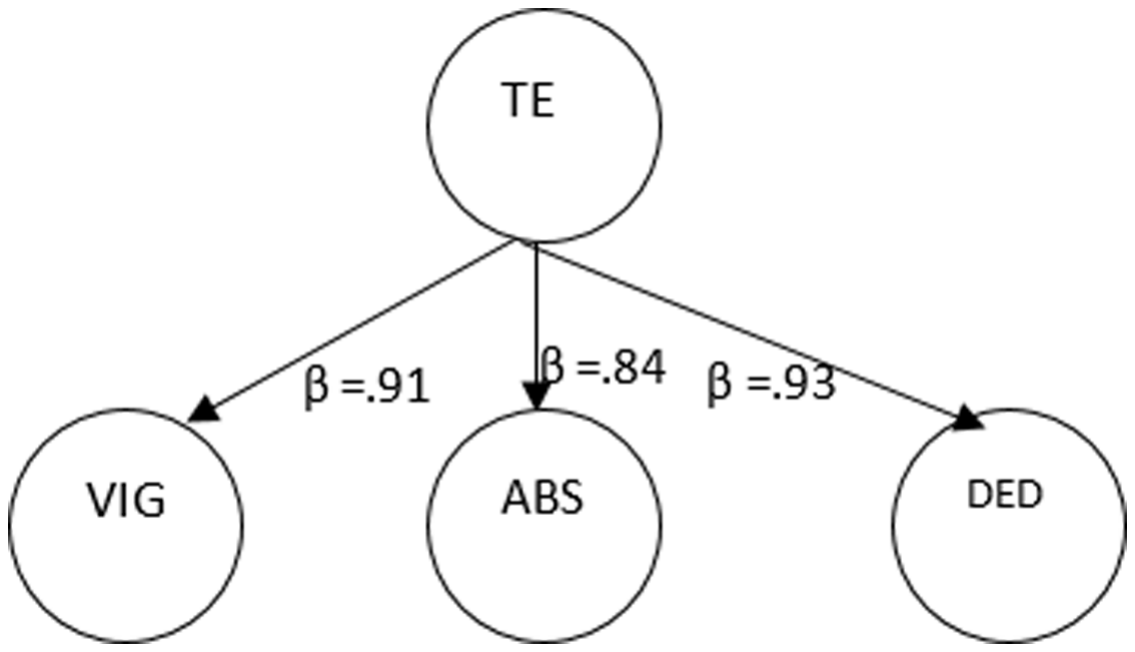
Second order factor analysis of team engagement.

Moreover, team engagement (TE) was also modeled as a second-order construct comprising three first-order dimensions: vigor (VIG), absorption (ABS), and dedication (DED). As shown in Figure 5, the standardized loadings of VIG, ABS, and DED on TE exceeded 0.60. This result suggests that TE, as a three-dimensional second-order construct, is significantly represented by vigor, absorption, and dedication.

**Figure 6:**
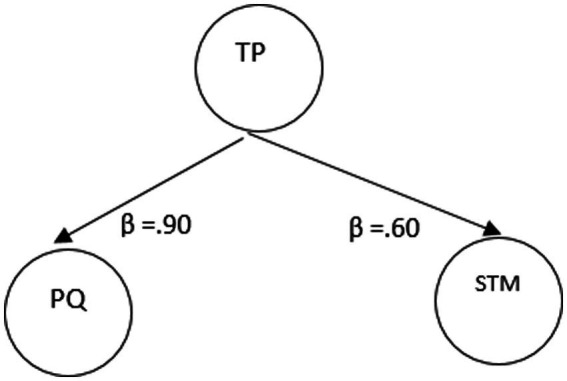
Second order factor analysis of team performance.

Finally, team performance (TP) was modeled as a second-order construct comprising two first-order dimensions: product quality (PQ) and speed to market (STM). Figure 6 shows the standardized regression loadings of these two constructs. As shown in Figure 6, both constructs were close to or exceeded the recommended standardized loading threshold of 0.60. This result suggests that team performance, as a two-construct second-level variable, is significantly represented by product quality and speed to market.

### 3.5 Assessment of common method variance

In order to check for common method variance (CMV), we followed Kock (2015). Kock (2015) proposed that a model with greater than 3.3 variance inflation factors (VIFs) is an indication of a CMV issue. The results of our VIFs analysis demonstrate that the VIFs values range between 2.62 and 2.95, i.e., the values are lower than the suggested threshold of 3.3. Thus, the proposed model does not appear to be affected by common method bias.

### 3.6 Hypothesis testing

The Partial Least Squares (PLS) approach (Ringle et al., 2005) combined with the bootstrapping resampling method (Chin, 2001) was employed using the SmartPLS 3.0 software to test the hypotheses and evaluate the predictive power of the proposed model (see Figure 1). This process involved generating 5,000 sub-samples of cases (Hair and Ringle, 2013), which were randomly selected with replacement from the original dataset. Path coefficients were then calculated for each sub-sample. T-statistics were subsequently computed for all coefficients, based on their consistency across these sub-samples, to identify statistically significant relationships. The resulting path coefficients and their associated t-values indicated the strength and direction of each hypothesized relationship.

Table 5 presents the results of hypothesis testing, detailing the path coefficients (*β*), significance levels (*p*-values), and outcomes for each hypothesis. The analysis examines the relationships among e-leadership, team emotional capability, team engagement, and team performance.

**Table 5:** Path results.

Path	β	*P*	H	Result
E-L → TP	−0.253	0.352	H1	Not supported
E-L → TEC	0.805**	0.000	--	--
E-L → TE	0.022	0.223	--	--
TEC → TE	0.615**	0.000	--	--
TEC → TP	0.639**	0. 000	--	--
TE → TP	0.040	0.835	--	--

**: *p* < 0.00. Note: E-L: E-leadership, TEC: Team emotional capability, TE: Team engagement, TP: Team Performance.

The findings indicate that e-leadership’s effect on team performance is not significant (β = −0.253, *p* > 0.05), indicating no support for H1. This result does not mean that e-leadership is not related to team performance, its direct influence on performance outcomes may be limited or mediated by factors such as team engagement or emotional capability.

Moreover, regarding the direct effects, we find that e-leadership has a significant positive effect on team emotional capability (β = 0.805, *p* < 0.01) highlighting that e-leadership plays a crucial role in fostering team emotional capability by enhancing collaboration, emotional awareness, and mutual understanding among team members. In contrast, the relationship between e-leadership and team engagement is not statistically significant (β = 0.022, *p* > 0.05), suggesting that while e-leadership may indirectly influence engagement through other factors like team emotional capability, its direct effect on engagement is minimal.

Furthermore, team emotional capability significantly impacts team engagement (β = 0.615, *p* < 0.01). This finding underscores the importance of emotional capacity in driving team engagement, as emotionally capable teams are more likely to demonstrate dedication, vigor, and focus. Additionally, team emotional capability has a significant positive effect on team performance (β = 0.639, *p* < 0.01), suggesting that emotional capability is a key determinant of team performance, likely by enabling effective communication, collaboration, and alignment toward shared objectives.

Finally, the relationship between team engagement and team performance is not statistically significant (β = 0.040, *p* > 0.05), indicating that while engagement reflects positive team behaviors, its direct impact on performance may depend on additional mediating variables, such as team emotional capability or leadership styles (Table 5).

Table 6 reports the mediation results examining whether team emotional capability (TEC) transmits the effects of e-leadership (E-L) to team performance (TP) and team engagement (TE), and whether a sequential pathway (E-L → TEC → TE → TP) is supported. VAF is reported only for paths with directionally consistent direct and indirect effects; where sign inconsistency is observed, VAF is omitted because it may fall outside the 0–100% range and is not interpretable as a “share of mediation” (Table 6, note a).

**Table 6:** Results for the mediation analysis.

Total effect		Direct effect		Indirect effect		
Relationship	β	Relationship	β	Relationship	β	VAF (%)
E-L→TP	0.290*	E-L→TP	−0.253	E-L→TEC→TP	0.514**	---^a^
E-L→TE	0.697**	E-L→TE	0.022	E-L→TEC→TE	0.495**	71
E-L→TP	0.290*	E-L→TP	−0.253	E-L→TEC→TE→TP	0.020	---^a^

*: *p* < 0.05, **: *p* < 0.00. E-L: E-leadership, TEC: Team emotional capability, TE: Team engagement, TP: Team Performance experience.

a Given the sign inconsistency between the direct and indirect effects, VAF is omitted for this path. In such cases, VAF may yield values beyond the 0–100% range and should not be interpreted as the share of the total effect explained by the indirect effect.

For team performance, the total effect of e-leadership is positive and significant (β = 0.290, *p* < 0.05). In contrast, the direct effect is negative and non-significant (β = −0.253, *p* > 0.05), while the indirect effect via TEC is positive and significant (β = 0.514, *p* < 0.001). This configuration indicates a sign inconsistency between direct and indirect pathways, consistent with inconsistent mediation (suppression), where the positive indirect pathway through TEC offsets (and may mask) the negative direct component (MacKinnon et al., 2000). Accordingly, VAF is not reported for this relationship (Table 6). Substantively, these results suggest that the beneficial influence of e-leadership on team performance operates primarily through enhancing teams’ emotional capability, supporting the mediating role of TEC for this linkage.

For team engagement, the total effect of e-leadership is positive and significant (β = 0.697, *p* < 0.01). When TEC is modeled as a mediator, the direct effect becomes non-significant (β = 0.022, *p* > 0.05), whereas the indirect effect via TEC remains positive and significant (β = 0.495, *p* < 0.001). The VAF value (71%) indicates that a substantial proportion of the overall association between e-leadership and team engagement is explained through TEC, providing strong evidence that TEC is a key transmission mechanism linking e-leadership to engagement outcomes.

Finally, the sequential indirect effect from e-leadership to team performance through TEC and TE is positive but non-significant (β = 0.020, *p* > 0.05). Given that the direct effect on TP is negative while the sequential indirect effect is positive, this pathway also exhibits sign inconsistency, and VAF is therefore omitted (Table 6, note a). Collectively, these findings do not support the sequential mediation hypothesis; rather, they indicate that TEC functions as the primary mediator in the model, robustly linking e-leadership to team engagement and contributing to team performance, while the additional step through team engagement does not provide a statistically meaningful incremental mechanism for explaining team performance.

### 3.7 Structural model

Table 7 presents a detailed evaluation of the structural model using the Partial Least Squares Structural Equation Modeling (PLS-SEM) approach, focusing on key metrics such as the coefficient of determination (R^2^), predictive validity (Q^2^), and the standardized root mean squared residual (SRMR). These indicators are crucial for assessing both the model’s explanatory power and its predictive validity for the endogenous constructs.

**Table 7:** Structural model metrics.

Endogenous constructs	R^2^	Q^2^	SRMR
TEC	0.648	0.291	0.092
TE	0.619	0.332	
TP	0.240	0.061	

The R^2^ values indicate the proportion of variance in the dependent constructs explained by the predictors in the model. Following Chin’s (2001) guidelines, R^2^ values are classified as small (0.02 to 0.13), medium (0.13 to 0.26), or large (0.26 and above). In this study, the R^2^ value for Team Emotional Capability (TEC) is 0.648, representing a large effect size and indicating that the model explains a substantial proportion of the variance in TEC. The R^2^ for Team Engagement (TE) is 0.619, also signifying a large effect size, while the R^2^ value for Team Performance (TP) is 0.240, reflecting a medium effect size. Collectively, these R^2^ values demonstrate that the structural model has strong explanatory power, particularly for TEC and TE.

The Q^2^ values, which assess the predictive validity of the model, confirm its ability to predict the data points of the dependent constructs. Positive Q^2^ values across all constructs indicate that the model has predictive relevance. Specifically, TEC has a Q^2^ value of 0.291, indicating moderate predictive validity. The Q^2^ value for TE is 0.332, suggesting strong predictive validity, while the Q^2^ value for TP is 0.061, showing a weaker but still positive predictive relevance. These results affirm the model’s predictive capabilities, particularly for TEC and TE.

The SRMR value, which evaluates the model’s fit, is reported as 0.092. According to Hu and Bentler (1998), an SRMR value below 0.08 indicates a good fit, while Kock (2020) suggest that an SRMR value below 0.10 is acceptable in the PLS-SEM context. Hence, the SRMR value of 0.092 suggests that the model exhibits an acceptable level of fit to the data (Table 7).

Overall, the structural model demonstrates strong explanatory and predictive power. The high R^2^ values for indicate the model’s robustness in explaining these constructs, while the positive Q^2^ values reinforce its predictive capabilities. Although the SRMR value exceeds the stricter threshold of 0.08, it remains within the acceptable range for PLS-SEM, affirming the model’s adequacy in explaining the relationships between e-leadership, emotional capability, team engagement and team performance.

## 4 Discussion

This study set out to examine the role of e-leadership in distributed software development teams by exploring its impact on team performance through the mediating mechanisms of team emotional capability and team engagement. The findings provide valuable insights into how the indirect effects of e-leadership, specifically its capacity to enhance team emotional capability, play a pivotal role in driving team engagement and ultimately translating into improved performance in distributed work settings.

Our analysis reveals that there is no significant direct relationship between e-leadership and team performance. This suggests that, in isolation, e-leadership does not immediately enhance performance outcomes. Instead, its effectiveness appears to hinge on its capacity to shape underlying team processes, a finding that echoes previous research demonstrating that leadership influences are often mediated by other factors rather than exerting a direct, observable effect (Podsakoff et al., 1990; Avolio et al., 2014).

Interpreting this pattern through social exchange theory, the absence of a direct effect is not unexpected: ICT-mediated leader behaviors are relatively distal to software-based NPD outcomes and typically influence performance only after they are converted into shared relational resources (e.g., team emotional capability) and coordinated action. Moreover, ICT-intensive collaboration may entail exchange costs—such as information overload and technostress—that can absorb attention and increase coordination fatigue in virtual teams, potentially attenuating short-term performance links. Although we did not directly measure these costs, prior research (e.g., Eppler and Mengis, 2004; Ellwart et al., 2015) suggests that ICT-enabled work can generate overload and strain that may coexist with (and partly mask) positive indirect effects. This combination aligns with masking/indirect-only mediation logic (MacKinnon et al., 2000; Zhao et al., 2010) and underscores that e-leadership effectiveness in distributed software NPD teams should be evaluated via proximal team processes rather than direct outcome links.

Conversely, the study demonstrates that team emotional capability serves as a critical mediator between e-leadership and team performance. When e-leaders effectively cultivate their teams’ ability to perceive, regulate, and leverage emotions, the resulting emotional intelligence facilitates superior performance outcomes. This finding corroborates earlier studies (Akgün et al., 2011; Holtz et al., 2020) and reinforces the view that in the absence of face-to-face interaction, emotional management is essential for bridging leadership behaviors and team performance. Moreover, scholars such as Curşeu et al. (2015) and Aritzeta et al. (2020) have argued that teams with heightened emotional capability are better equipped to navigate the complexities of remote collaboration, leading to improved communication, conflict resolution, and overall performance (Côté and Miners, 2006; Farh et al., 2012).

Additionally, our findings underscore that team emotional capability fully mediates the relationship between e-leadership and team engagement. In distributed settings, where non-verbal cues and physical proximity are limited, the development of emotional capability is paramount for sustaining engagement (Kashive et al., 2022; Aritzeta et al., 2020). High levels of emotional capability enable teams to engage in positive emotional exchanges, thereby fostering an environment of commitment and collaboration (Koutsioumpa, 2023; Huang, 2022; Carasco-Saul et al., 2014; Mazzetti, 2022). Conversely, teams lacking this capacity may experience unresolved emotional conflicts that hinder effective engagement and ultimately impair performance (Sun et al., 2021; Wit et al., 2012).

Notably, the sequential mediation model, postulating a pathway from e-leadership through team emotional capability to team engagement and ultimately to team performance, was not supported. One plausible explanation is that in distributed work settings, tasks are often executed in a more individualized manner, which impedes the formation of genuine team unity. Even when individual engagement is high, the absence of coordinated, collective interaction may limit its translation into overall team performance (Gilson et al., 2015; Contreras et al., 2020). This finding reinforces the notion that, in technology-mediated contexts, the immediate enhancement of team emotional processes is more critical for performance than the subsequent development of engagement (Rezvani et al., 2018; Contreras et al., 2020).

An additional noteworthy aspect of these findings is the presence of a masking mediation effect. Although the direct path from e-leadership to team performance was negative and non-significant, the strong positive indirect path through team emotional capability compensated for and obscured this relationship. This suggests that without accounting for mediating mechanisms, one might prematurely conclude that e-leadership has no effect on performance. Similar suppression or masking effects have been documented in leadership–performance research, where indirect pathways reverse or conceal direct effects (Kim and Beehr, 2017; Li et al., 2017; Nohe and Hertel, 2017; Malik et al., 2025). In reality, the distributed context amplifies the salience of emotional capability, making it the critical conduit through which e-leadership exerts its influence. Thus, the value of e-leadership in technology-mediated environments lies less in direct managerial actions and more in its capacity to build collective emotional competence that transforms digital coordination into sustainable performance outcomes.

### 4.1 Theoretical contributions

This research makes three primary contributions to the e-leadership and virtual team literatures. First, it clarifies the mechanism linking e-leadership to distributed team performance by demonstrating that team emotional capability is the central pathway through which ICT-mediated leadership is translated into superior outcomes. Rather than treating e-leadership as directly performance-enhancing, our findings suggest that leaders create value by cultivating collective affective resources that enable members to coordinate, learn, and recover from setbacks in digitally mediated collaboration.

Second, the study enriches SET by showing that exchange quality in distributed work is not only a dyadic phenomenon but also accumulates into team-level emotional capability that shapes how reciprocity is enacted under reduced cues and physical distance. In this sense, team emotional capability functions as a socioemotional “exchange resource” that sustains commitment, mutual helping, and coordinated effort when work is performed through digital media.

Third, by focusing on distributed NPD teams, the study highlights the contextual importance of emotional capability in innovation work characterized by uncertainty, iteration, and time pressure. This NPD emphasis helps bridge the e-leadership literature with product innovation and project research by identifying a team-level affective capability that is especially consequential for quality and speed-to-market outcomes.

### 4.2 Practical implications

The results of this study demonstrate that e-leadership effectiveness in distributed NPD teams does not originate from the intensity of digital communication alone, but from the leader’s capability to deliberately convert technology-mediated interaction into shared emotional resources that sustain coordination and learning. Accordingly, organizations should design induction and development programs for new e-leaders that combine digital literacy (tool selection, media-fit decisions, asynchronous coordination, digital etiquette) with relational competencies (trust building, conflict navigation, and emotion-focused communication in low-cue settings).

From a managerial standpoint, this implies that leaders of distributed NPD teams should establish routines that directly support the development of team emotional capability. Examples include: sprint-level emotional check-ins in stand-ups and retrospectives; explicit decision logs and transparent priority boards to reduce uncertainty; structured conflict-resolution protocols for remote disagreements; and psychologically safe channels for surfacing concerns before they escalate. These practices help teams recognize and regulate collective emotions during iteration, rework, and deadline pressure, thereby protecting quality and speed-to-market.

Organizations should also adapt their human resource systems to reflect these insights. Recruitment, selection, and appraisal criteria can emphasize leaders’ demonstrated ability to build team emotional capability (e.g., fostering openness, reconciliation after conflict, and shared identification), not merely technical oversight. Complementary interventions such as team charters, peer coaching, and short workshops on emotion regulation and feedback in remote collaboration can accelerate the development of team-level emotional capability across NPD teams.

### 4.3 Limitations and future research

While this study advances our understanding of e-leadership in distributed work contexts, several limitations warrant discussion. First, a primary concern is common method bias, a well-known challenge in cross-sectional survey research (Podsakoff et al., 2003). To mitigate measurement biases associated with this issue, we employed a multi-source data collection strategy. Specifically, data for the predictor (i.e., e-leadership) and mediator (i.e., team emotional capability and team engagement) variables were gathered from team members, whereas team performance data were obtained independently from team leaders. In addition, we applied Kock’s (2015) procedure to further assess and address common method bias, as detailed in the methodology section. Finally, to overcome the inherent limitations of retrospective data collection, we implemented a free reporting method (Huber and Power, 1985), allowing participants the option to omit responses to any items with which they were uncomfortable, as outlined in the consent form.

Second, our sample is limited to software development teams in technology parks within Istanbul and Kocaeli. This specialized context may restrict the generalizability of the findings to other industries, regions, or cultural settings (Podsakoff et al., 2003). Future research should replicate these findings across diverse sectors and geographic areas to confirm the robustness of the observed relationships.

Third, the cross-sectional design of this study limits causal inferences. Longitudinal studies would be valuable in elucidating the temporal dynamics between e-leadership, team emotional capability, and performance outcomes (Avolio et al., 2014). Such an approach could yield deeper insights into the evolution and sustainability of e-leadership effects in distributed teams.

Fourth, although team emotional capability emerged as a key mediator, other potential mediators or moderators, such as team size, task interdependence, or cultural dimensions, may also influence the relationship between e-leadership and team performance (Graen and Uhl-Bien, 1995). Future investigations should explore these additional variables to develop a more comprehensive model of e-leadership in virtual work environments. Additionally, while leader–member exchange (LMX) theory informed parts of our conceptual framing, we did not directly measure LMX relationship quality or differentiation; future research should include LMX measures to test how dyadic exchange patterns aggregate to team-level capabilities in distributed NPD.

Finally, the nonsignificant sequential mediation involving team engagement suggests that the translation of individual engagement into collective performance may be impeded by the individualized nature of distributed work. In such settings, even high individual engagement may not culminate in cohesive team performance due to a lack of coordinated interaction (Contreras et al., 2020). Future research should further examine how team engagement interacts with emotional capability and other contextual factors to influence performance outcomes.

## 5 Conclusion

In conclusion, this study contributes to the evolving discourse on e-leadership in distributed teams by demonstrating that the benefits of e-leadership are primarily transmitted through enhanced team emotional capability. Although e-leadership does not directly boost team engagement or performance, its ability to strengthen the emotional fabric of teams is central to fostering collaborative, resilient, and high-performing virtual work environments. For practitioners, these insights underscore the necessity of prioritizing emotional intelligence and digital communication strategies in leadership development. For scholars, the findings open new avenues for exploring the intricate interplay of leadership, emotion, and team dynamics in an increasingly digital and dispersed work landscape.

## Data Availability

The raw data supporting the conclusions of this article will be made available by the authors, without undue reservation.
