# Supplementary material for: E-leadership in distributed new product development teams: unveiling the interplay between performance, team emotional capability, and engagement
Source: Front Psychol. 2026 Jun 16;17:1802949. doi: 10.3389/fpsyg.2026.1802949 (PMC13314413; doi:10.3389/fpsyg.2026.1802949)
Supplement: Supplementary file 1 [file Data_Sheet_1.docx]

**Appendix**

Appendix Measure vision-inspiring role model, fostering acceptance of group goals, intellectual stimulation, individualized support, and high-performance expectations

Standardized loadings are in parentheses.

* denotes the dropped item; either they reduce the AVE to less then .50, or they have low loading weights.

**E-LEADERSHIP**

**E-Communications Skills Adopted from Van Wart et al. (2019)**

1. Actively engages with team members on online platforms to foster collaboration and facilitate the sharing of knowledge. (0.781)

2. Develops and disseminates relevant content through various social media channels to promote the team's accomplishments and efforts. (0.782)

3. Monitors social media feedback and comments, ensuring timely and appropriate responses. (0.804)

4. Leverages social media to remain informed about industry trends and developments, sharing insights with team members to support continuous learning and professional growth. (0.799)

r_wg_: 0.682

**Vision-Inspiring Role Model Adopted from Podsakoff et al. (1990)**

1. Has a clear understanding of where we are going. (0.827)

2. Paints an interesting picture of the future for our group. (0.829)

3. Is always seeking new opportunities for the organization. (0.822)

4. Inspires others with his/her plans for the future. (0.843)

5. Is able to get others committed to his/her dream. (0.713)

6. Leads by “doing,” rather than simply by “telling.” (0.810)

7. Provides a good model for me to follow. (0.777)

8. Leads by example. (0.795)

r_wg_: 0.714

**Fostering Acceptance of Group Goals Adopted from Podsakoff et al. (1990)**

1. Fosters collaboration among work groups. (854.)

2. Encourages employees to be “team players.” (0.873)

3. Gets the group to work together for the same goal. (0.900)

4. Develops a team attitude and spirit among employees. (0.845)

r_wg_: 0.843

**Individualized Support Adopted from Podsakoff et al. (1990)**

1. Considers my feelings before taking action. (0.846)

2. Shows respect for my personal feelings. (0.887)

3. Behaves in a manner thoughtful of my personal needs. (0.870)

4. Treats me with consideration for my personal feelings. (0.833)

r_wg_: 0.748

**Intellectual Stimulation Adopted from Podsakoff et al. (1990)**

1. Challenges me to think about old problems in new ways. (0.802)

2. Asks questions that prompt me to think. (0.796)

3. Has stimulated me to rethink the way I do things. (0.856)

4. Has ideas that have challenged me to reexamine some of my basic assumptions about my work. (0.784)

r_wg_: 0.815

**High-Performance Expectations Adopted from Podsakoff et al. (1990)**

1. Shows us that he/she expects a lot from us. (0.754)

2. Insists on only the best performance. (0.799)

3. Will not settle for second best. (0.813)

r_wg_: 0.798

**TEAM EMOTIONAL CAPABILITY (adopted from Akgün et al., 2007 and Akgün et al., 2011)**

**Dynamics of Encouragement**

1. Our team has an ability to instill hope among all its members (0.891).

2. Managers in our team encourage enthusiasm (0.909).

3. Managers in our team foster a work environment that encourage employees to exhibit courage in their actions (0.816=

4. Managers in our team infuse hope and joy in the team (0.655).

r_wg_: 0.729

**Dynamics of Display of Freedom**

1. Our team has the ability to facilitate the variety of authentic emotions that legitimately can be displayed (0.919).

2. In our team, people are encouraged to express their full range of emotions without fear of reprisal (0.929).

r_wg_: 0.815

**Dynamics of Playfulness**

1. Our team creates a context that encourages experimentation (0.746).

2. Our team tolerates mistakes of people who take initiatives (0.776).

3. In our team, a safe and protective work environment is created to test new team identities (such as new processes or ideas) without premature lock-in (0.889).

r_wg_: 0.756

**Dynamics of Identification**

1. Members of our team express their deep attachment to salient team characteristics such as values and beliefs (0.876).

2. Members of our team stay together because there are mutual benefits, among which emotional bonds developed over time are the most important (0.899).

3. People defend our team’s name and reputation beyond work boundaries (0.747).

r_wg_: 0.851

**Dynamics of Experiencing**

1. Our team members have the ability to understand others’ feelings (0.879).

2. People in our team experience the same or other appropriate emotions in response to others’ feelings (0.745).

3. People in our team communicate their emotions with others (0.842).

4. People are able to read the subtle social cues and signals given by others to determine what emotions are being expressed and understand the perspective of other individuals (0.899).

5. In our team, efforts are made to uncover and understand the emotions held by other members.(0.903)

r_wg_: 0.722

**Dynamics of Reconciliation**

1. Our team has the ability to bring together two seemingly opposing values that people feel strongly about (0.861).

2. People in our team retain their private feelings while understanding those of others. (0.849).

3. People in our team can jointly develop a meaningful bridge between their various emotions (0.822).

4. People in our team feel the general emotion of another without directly sharing that person’s experience (0.816).

5. People in our team can maintain their feelings while appreciating those of others*

r_wg_: 0.888

**TEAM ENGAGEMENT SCALE Adapted from Costa et al. (2014)**

**Vigor**

1. At our work, we feel bursting with energy (0.870)

2. At our job, we feel strong and vigorous (0.867)

5. When we arrive at work in the morning, we feel like starting to work (0.779)

r_wg_: 0.815

**Dedication**

3. We are enthusiastic about our job (0.929)

4. Our job inspires us (0.844)

7. We are proud on the work that we do (0.832)

r_wg_: 0.873

**Absorption**

6.We feel happy when we are working intensely (0.837)

8. We are immersed in our work (0.816)

9. We get carried away when we are working (0.761)

r_wg_: 0.799

**TEAM PERFORMANCE SCALE**

**Product Quality adopted from Atuahene-Gima et al. (2006).**

1. The quality of the new product (software) compared well with competing products. (0.754)

2. The quality of the new product (software) was higher than competitor products. (0.799)

3. The quality of the new product (software) was better than other products of the firm. (0.813)

4. The new product (software) was perceived by customers as very reliable with respect to competing products. *

5. Customers perceived the new product (software) to be better than the competition. (0.645)

r_wg_: 0.623

**Speed to Market adopted from Kessler and Chakrabarti, (1999)**

1. This product (software) was developed and launched (fielded) faster than the major competitor for a similar product (.926)

2. This product (software) was completed in less time than what was considered normal and customary for our industry (.843)

3. This product (software) was launched on or ahead of the original schedule developed at initial project go ahead (.865)

r_wg_: 0.817
